# Primary acinic cell carcinoma of the breast: A case report with a clinicopathological and immunohistochemical study of a rare breast cancer subtype

**DOI:** 10.1016/j.amsu.2018.09.030

**Published:** 2018-09-27

**Authors:** Rajeev Sen, Namita Bhutani, Jashanpreet Kamboj, Sakshi Dahiya

**Affiliations:** Deptt. of Pathology, PGIMS Rohtak, Haryana, India

**Keywords:** Acinic cell carcinoma of breast, Case report, Immunohistochemistry, Morphology, Triple-negative breast cancer

## Abstract

Acinic cell carcinoma (ACC) of the breast is extremely rare subtype of triple-negative breast carcinoma and demonstrates extensive morphologic overlap with acinic cell carcinoma of the salivary gland. Herein, we report a case of acinic cell carcinoma of the breast in a 41-year old female presenting with a palpable breast mass along with significant morphological and immunohistochemical findings. Histologically, ACC showed a diffuse glandular infiltrative pattern, with small acinar or glandular structures mixed with solid nests. Both glandular and solid tumor cell populations were strongly positive for lysozyme. The immunohistochemical profile of the tumor was also similar to that of salivary gland acinic cell carcinoma. She received postoperative chemoradiation therapy and has been doing well. As studies on large series are lacking, further studies are needed to elucidate the biological characteristics of acinic cell carcinoma of the breast. In this study, we perform a detailed morphologic and immunohistochemical description of cases of this rare entity and undertake a comprehensive review of all reported cases of breast acinic cell carcinoma in the English language literature to date.

## Highlights

• Acinic cell carcinoma of the breast is extremely rare subtype of breast carcinoma.
• Only 16 cases of ACC of the breast have been reported since 1996.
• Immunohistochemical examination is important in making an accurate diagnosis.

## 1 Introduction

Acinic cell carcinoma (AcCC) of breast was first described by Roncaroli et al. [1] in 1996 and is recognized as a subtype of triple-negative breast carcinoma (TNBC) in the current World Health Organization classification [2]. It is one of several rare subtypes of breast carcinoma that demonstrate morphologic overlap with the repertoire of tumors seen in the salivary glands [3]. Although most breast carcinomas are “ductal” in appearance and show no evidence of acinar or “secretory”-type differentiation, rare cases of invasive ductal carcinoma (IDC) can demonstrate S-100– or lysozyme-positive cells with granular cytoplasm [4], including carcinomas with apocrine morphology, or those that arise in microglandular adenosis (MGA) [5]. Although the term secretory carcinoma is currently used exclusively to describe breast carcinomas associated with the presence of the ETV6- NTRK3 translocation, it may be said that there is a larger subcategory of breast carcinomas, including rare entities such as AcCC and cystic hypersecretory carcinoma, which recapitulate the prosecretory phenotype of the lactating breast. Here, we report a recent case of breast AcCC diagnosed at our institution, and we review what is currently known about this rare entity in terms of morphology, immunohistochemistry, and molecular pathology. We state that the work has been reported in line with the SCARE criteria [6].

## 2 Case report

A 41-year-old female presented with a 7 months history of a palpable breast lump in right upper outer quadrant. No abnormalities were discovered on physical examination. Results of laboratory tests were all within the reference range. Sonography and mammographic findings showed no evidence of abnormality in either breast or axillae. The Fine Needle Aspiration (FNA) was atypical cytology, and showed scattered or rarely clustered, uniformly round cells with small nuclei and a moderate amount of cytoplasm, suggesting a benign or low grade malignant tumor. Core needle biopsy (CNB) of the mass was diagnosed as Infiltrating Ductal carcinoma-NOS. Modified radical mastectomy was done and sent for histopathological examination.

**Fig. 1:**
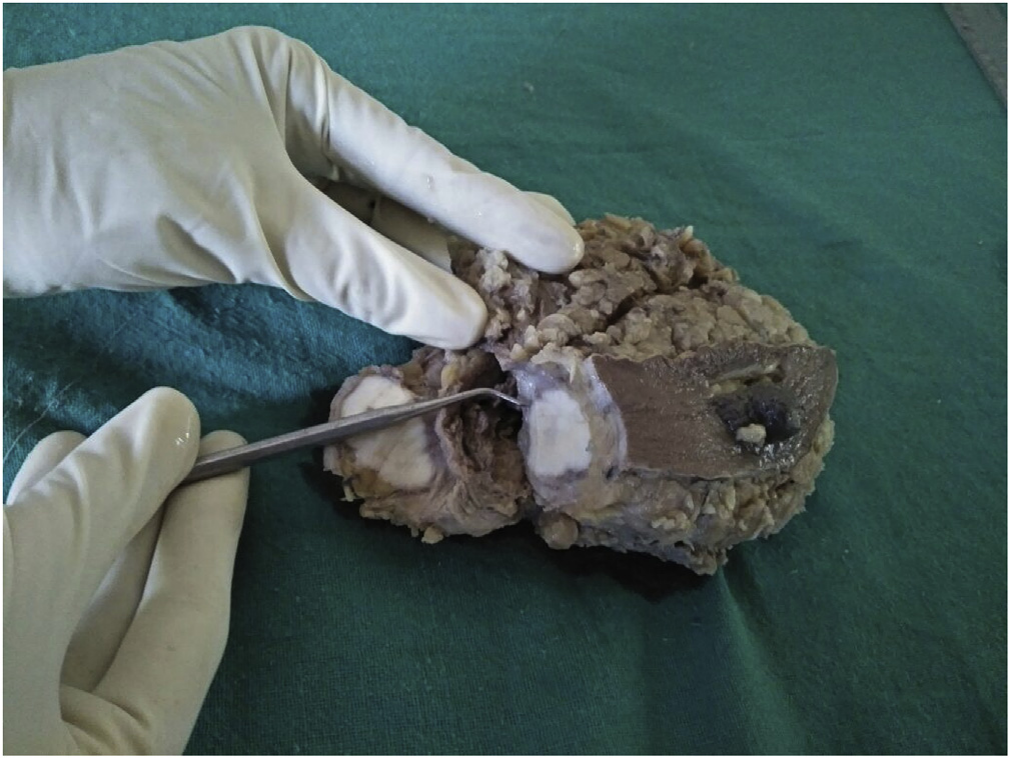
The tumor of size 2.5 × 2.0 × 1.0 cm, with a gray BROWN rubbery consistency and a slightly lobulated appearance. (For interpretation of the references to colour in this figure legend, the reader is referred to the Web version of this article.)

**Fig. 2:**
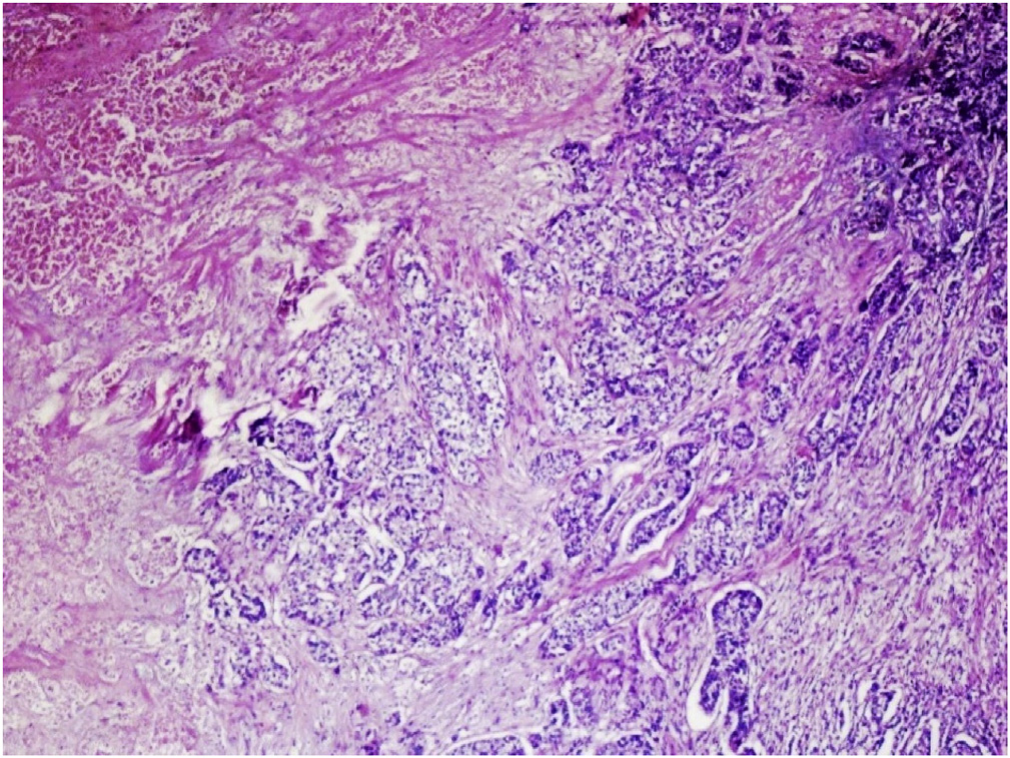
Tumor with diffuse infiltrative growth pattern with small acinar or glandular structures mixed with solid nests (40×).

**Fig. 3:**
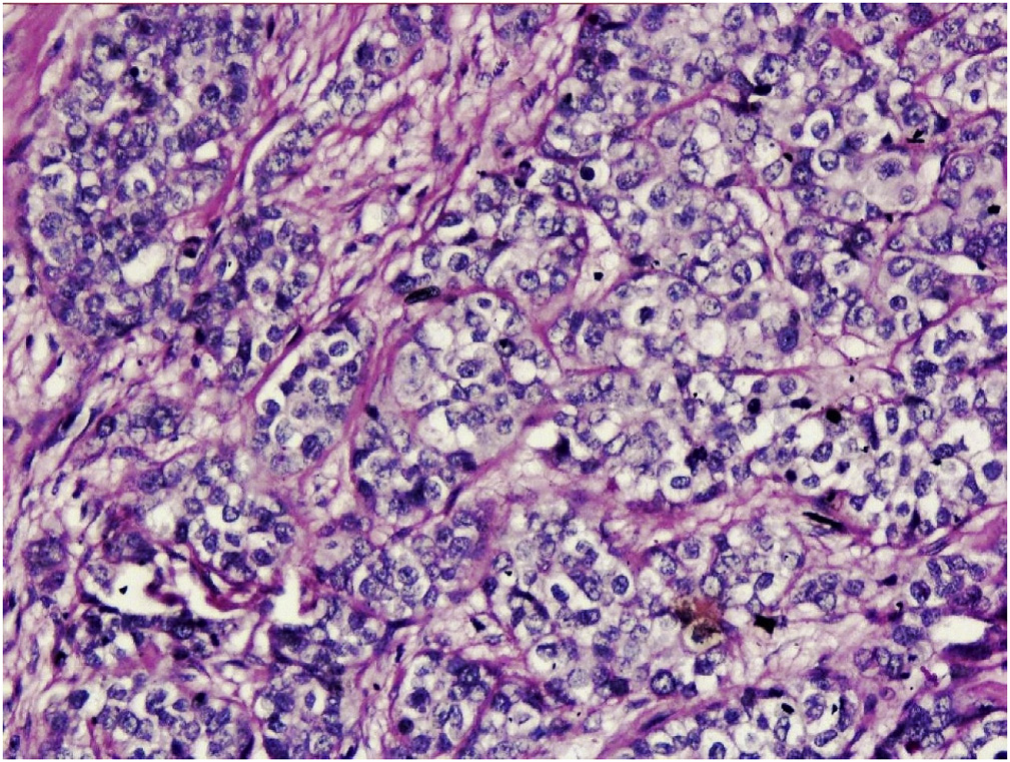
Monotonous round cells with a finely granular, weakly eosinophilic, or clearly vacuolated cytoplasm (H&E stain, × 200).

On gross examination, the tumor was a 2.5 × 2.0 × 1.0 cm, gray white with a rubbery consistency, with a well-defined border and a slightly lobulated appearance (Fig. 1). Microscopically, the tumor showed a diffuse infiltrative growth patterns with small acinar or glandular structures mixed with solid nests (Fig. 2, Fig. 3). Most of the tumor was comprised of monotonous round cells with a finely granular, weakly eosinophilic, or clear vacuolated cytoplasm resembling acinar cells of the salivary glands. Some neoplastic cells had a clear cytoplasm. The nuclear grade of the tumor cells was determined to be grade 2. Foci of vascular invasion were also present. Lymphatic permeation was occasionally seen, but the sentinel lymph node was free of metastasis. The mitotic count was up to 0–3/high power field. The Final surgical margins were negative. Cells with eosinophilic granules and globules were strongly periodic acid–Schiff (PAS) (diastase resistant) positive.

**Fig. 4:**
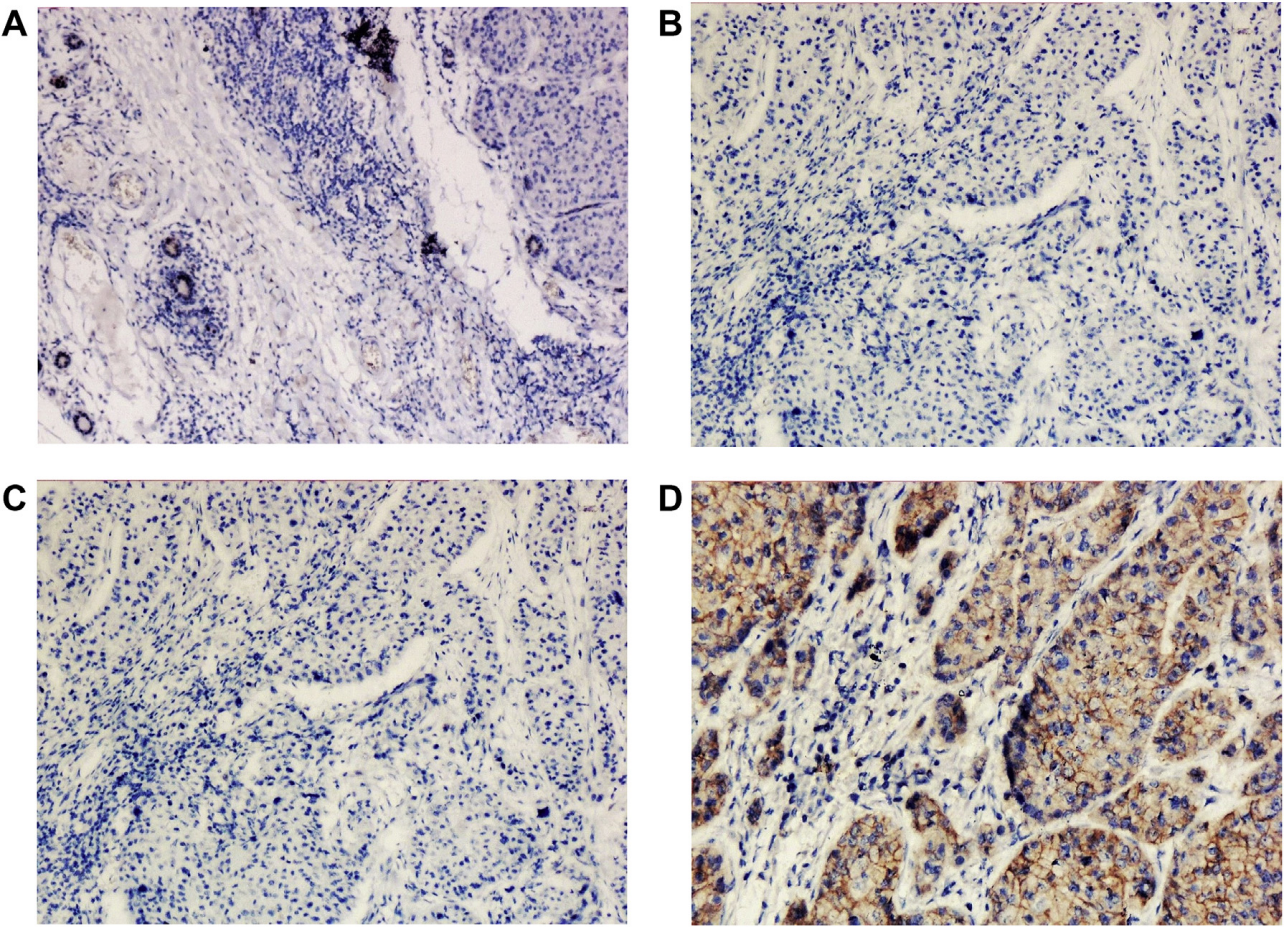
A: ER negative in tumor cells. B: PR negative in tumor cells. C: HER2 neu negativee in tumor cells. D: Lysozyme positive in tumor cells.

Immunohistochemically, tumor cell populations were strongly positive for lysozyme (Fig. 4A and D) and were negative for estrogen receptor, progesterone receptor and human epidermal growth factor receptor-2 (HER2/neu). Because the patient was diagnosed as having invasive breast cancer with a triple-negative phenotype, postoperative radiotherapy (50Gy-/25 fractions) followed by adjuvant chemotherapy (TC: docetaxel 75mg/m2 and cyclophosphamide 600 mg/m2 administered intravenously every 3 weeks for 4 cycles) was administered. Although the followup period to date has been short (1 year), there have thus far been no signs of recurrence.

## 3 Discussion

The breast is embryologically, morphologically, and functionally related to secretory glands of other sites, including the salivary glands. It is, therefore, unsurprising that breast carcinomas may recapitulate the appearance of tumors more commonly seen in the salivary glands, including adenoid cystic carcinoma, pleomorphic adenoma, adenomyoepithelioma, myoepithelioma, oncocytic carcinoma, and mucoepidermoid carcinoma [3,7]. Matoso et al. emphasized that breast tumors with salivary gland differentiation originate from a malignant transformation of terminal duct-lobular units with metaplastic changes [8]. Conversely, ETV6-NTRK3 translocation–associated secretory carcinoma of the breast (T-SC) has a rare analogous counterpart in the salivary gland, mammary analog salivary gland carcinoma, which bears the same translocation [9]. However, T-SC is only one of the breast carcinoma subtypes that can demonstrate a “secretion-rich” phenotype, with variably sized cysts and pools of extracellular secretions and/or prominent cytoplasmic zymogen-like granules. These entities may together constitute a spectrum of morphologies in which the degree of development of the “secretory” phenotype varies from focal to predominant. Cystic hypersecretory carcinoma, with its dominant secretion-rich morphology, may be thought of as representing one end of this spectrum, whereas ductal carcinoma with focal eosinophilic granular cells [4] or pseudolactational features may represent the opposite pole of this morphologic range. The middle ground in this morphologic spectrum is represented by AcCC of breast. This entity has a wide morphologic spectrum of appearances in terms of architectural and cytologic features. Extensive intratumoral morphologic variation can be seen in a single tumor, highlighting the diagnostic challenges associated with core biopsy of these lesions.

Microscopically, ACC of the breast reveals two morphologically distinct cell populations focally merging into one another [10,11]. The present tumor showed a diffuse infiltrative growth pattern with small acinar or glandular structures mixed with solid nests. The solid nests, commonly observed in *in situ* and invasive ductal carcinoma, were focally recognized in the present case. Both glandular and solid tumor cell populations were strongly positive for lysozyme. The tumor cells were negative for estrogen receptor, progesterone receptor, and HER2/neu. Re-evaluation of the present case led to a diagnosis of pure ACC, due to the immunohistochemical results.

The IHC profile of breast AcCC shares many features with AcCC of salivary gland, with frequent expression of S-100, lysozyme, amylase, and A1-ACT and PAS positivity in addition. Although most cases have been negative for hormone receptors and all have been negative for HER2, it is useful to note that rare cases have shown some expression of ER and PR. Where myoepithelial markers such as calponin and p63 have been tested in breast AcCC, they are consistently negative, confirming the true invasiveness of this tumor type. Interestingly, the basement membrane associated proteins, collagen IV and laminin have also been negative in most breast AcCC cases where they have been reported [10].

No more than 16 cases of ACC of the breast have been reported since it was first described in 1996 as a rare variant of breast carcinoma showing morphological features resembling those of salivary glands [1]. ACC of the breast affects women between 20 and 80 years of age (mean, 54.2 years; with a single case involving a male patient). It generally presents as a palpable nodule ranging from 2 to 6 cm in size although 1 case involved a nonpalpable mass that was only discovered by mammography [10]. Several studies have discussed the usefulness of diagnostic imagings for ACC, although their findings have sometimes differed [1,11,12]. For example, mammography showed a well-defined mass in some cases [1,11] but no abnormal findings in another [13]. Ultrasonography revealed an intracystic tumor in only male patient yet described [12]. Thus, at present; it seems that there are no specific imaging findings that characterize this tumor type. Findings regarding tumor spread are similarly inconsistent. Lymph node metastasis was observed in 4 cases, and 3 cases showed nodal involvement upon recurrence with additional local, liver, and lung metastases. Only 1 patient died of the tumor, suggesting a relatively favorable prognosis for this tumor type although followup was limited to a maximum of 10 years [14].

In general, breast carcinoma lacking HER2 and the estrogen and progesterone receptors (triple-negative breast cancer TNBC) is more aggressive than other disease subtypes [15,16]. In contrast, ACC of the salivary glands is said to be a low-grade malignant neoplasm [17]. Therefore, it seems that ACC of the breast has characteristics similar to those of salivary gland, even if it is of the TNBC subtype. Although 1 patient was previously reported to have died as a result of this tumor type, standard adjuvant chemotherapy for breast cancer might not be always necessary. Several studies have reported that sporadic TNBC shares clinical and pathological features with hereditary *BRCA1*-related breast cancers, and, more recently, a case of a *BRCA1* mutation carrier with an ACC of the breast was reported [18]. Therefore, further studies are necessary to determine the optimal therapeutic strategy for these tumors.

Many early case reports suggested, based on very small case series, that breast AcCC is likely to have a good prognosis [1,10]. However, it is clear that poorly differentiated TNBC can frequently be a component of breast AcCC and that recurrences and death from this disease do occur. It seems probable that prognosis is largely driven by the presence of the poorly differentiated component. Furthermore, there is limited evidence from cases involving surgical excision after neoadjuvant chemotherapy that although the poorly differentiated tumor component may be comparatively chemosensitive, the better differentiated acinar and microglandular carcinoma may be more chemoresistant [19].

## 4 Conclusion

ACC of the breast is a rare variant of breast carcinoma that has been suggested to have a good prognosis even though it is often of the TNBC subtype. Currently, there are no characteristic diagnostic imaging findings for this disease, and immunohistochemical examination is important in making an accurate diagnosis. Further studies are needed to elucidate the biological characteristics ofACCof the breast. Recent molecular analysis of this tumor subtype suggests that the most common mutations (TP53, PIK3CA) seen in IDC NOS also occur in breast AcCC. Thus, in contrast to other analogous breast and salivary gland tumor types, such as ETV6-NTRK translocation–related tumors or MYB-NFIB translocation–related adenoid cystic carcinomas of both sites, AcCC of breast and salivary gland appear to be genetically distinct, despite their morphologic similarities.

## Consent

Written informed consent was obtained from the patients’ guardians for publication of this case series and any accompanying images.

We declare that there are no conflicts of interest amongst the authors.

We also state that there was no source of funding for this study.

## Ethical approval

Not applicable as this is a case report.

## Sources of funding

None.

## Author contribution

Namita Bhutani: Study design, data collection and wrote the article.

Rajeev Sen: Did the final editing.

Jashanpreet Kamboj: Contributed the clinical part.

Sakshi Dahiya: Managed the investigative part.

## Conflicts of interest

None.

## Research registry number

Not applicable.

## Guarantor

Pradeep Kajal.

## Appendix A Supplementary data

SS SS
